# The development of a Cancer Pain Belief Modification Program for patients with oral cancer in China: a feasibility study

**DOI:** 10.1186/s12912-023-01372-z

**Published:** 2023-06-16

**Authors:** Rongna Wang, Xiaoyan Zheng, Xixi Su, Xiuyu Huang, Huangju Liu, Yulai Guo, Ji Gao

**Affiliations:** 1 The School of Nursing, Fujian Medical University, Fuzhou, No.1 Xueyuan Road, Shangjie, Minhou, Fujian China; 2 Department of Otolaryngology head and neck surgery, Xi Jing Hospital, Air Force Medical University, Changle West Road 127, Xi’an, Shaanxi 710032 China; 3 Oral and Maxillofacial Surgery, The First Affiliated Hospital of Fujian Medical University, Fuzhou, Fujian China

**Keywords:** Oral cavity squamous cell carcinoma, Pain belief, Nurse, Quality of life, Pain, Pain management

## Abstract

**Background:**

Acceptance-based pain management interventions have been receiving growing attention in cancer pain care. This study aimed to develop a cancer pain management program based on belief modification to improve the cancer pain experience of Chinese oral cancer survivors and to explore the acceptability and preliminary outcomes of the Cancer Pain Belief Modification Program (CPBMP).

**Methods:**

A mixed-methods approach was applied to develop and revise the program. The CPBMP was developed and revised using the Delphi technique, and its further improvement was explored with a one-group pre- and post-trial designed with a sample of 16 Chinese oral cancer survivors, and semi-structured interviews. Research instruments included Numeric Rating Scale (NRS), Chinese version of Illness Perception Questionnaire-Revised for Cancer Pain (IPQ-CaCP), and the University of Washington Quality of Life assessment scale (UW-QOL). Descriptive statistics, t-test, and Mann–Whitney U test were used to analyse the data. The semi-structured questions were analysed using content analysis.

**Results:**

The six-module CPBMP was endorsed by most experts and patients. The expert authority coefficient value was 0.75 in the first round of the Delphi survey and 0.78 in the second round. The “pain intense”, “negative pain beliefs” scores of pre- and post-testing decreased from 5.63 ± 0.48 to 0.81 ± 0.54 (*t* = -3.746, *p* < 0.001); from 140.63 ± 9.02 to 52.75 ± 7.27 (*Z* = 12.406, *p* < 0.001); and the “positive pain beliefs”, “quality of life” scores increased from 55.13 ± 4.54 to 66.00 ± 4.70 (Z = -6.983, p < 0.001); from 66.97 ± 15.01 to 86.69 ± 8.42 (*Z* = 7.283, *p* < 0.001). The qualitative data also indicated that CPBMP was well acceptable.

**Conclusion:**

Our study showed the acceptability and preliminary outcomes of CPBMP patients. CPBMP improves the pain experience of Chinese oral cancer patients and provides a reference for cancer pain management in the future.

**Trial registration:**

The feasibility study has already been registered on the Chinese Clinical Trial Registry (ChiCTR) (www.chictr.org.cn) in 11/09/2021. (ChiCTR2100051065).

## Background

Oral cancer is the most common malignant tumor of the head and neck, including lip cancer, tongue cancer, gum cancer and floor of mouth cancer, which ranks sixth in global cancer incidence [1]. The incidence of oral cancer in China is on the rise year by year [2]. Oral malignant tumors are often in superficial locations and are important functional sites with very sensitive sensation, so patients experience severe cancer pain early [3]. The incidence of pain in patients with oral cancer was 88.4% [4]. Oral cancer patients have mild to moderate pain after surgery and during radiotherapy [5]. It has been found that pain severely impairs the speech, swallowing and chewing functions of oral cancer patients and has become a major concern for oral cancer survivors [5].

In 2020, the IASP revised the definition of pain, re-emphasizing that pain is an unpleasant sensory and emotional experience associated with, or resembling that associated with, actual or potential tissue damage [6]. Thus, pain is not only a sensation but also an emotional expression, which can be influenced by a combination of perceptual, emotional, and cognitive factors [7]. Pain beliefs belong to the category of pain cognition, which are individuals’ feelings, perceptions, and expected thoughts about the pain they experience [8]. Studies [9, 10] have confirmed that pain beliefs are significantly related to the duration of pain and pain intensity, and play a decisive role in pain directly or indirectly. The correlation between pain beliefs and pain levels in oral cancer patients was confirmed [9, 10]. Therefore, pain research should not be limited to the physiological and pathological changes of patients, but should delve into the influence of pain beliefs on pain in the pain cognitive level [10]. Pain beliefs are relatively stable and serve to help individuals make sense of the events they are experiencing or will experience [11]. Pain beliefs can be divided into negative pain beliefs and positive pain beliefs from a social conditioning level [12]. Negative pain beliefs mainly include fear, helplessness, and catastrophizing beliefs, while positive pain beliefs mainly include self-efficacy and control beliefs [12]. A study [13] found that oral cancer patients were most likely to have catastrophizing beliefs that “the pain will continue (they will have to endure pain for a long time)”. According to the findings [14], “stoicism” is a characteristic of “Chinese” pain, and more Chinese patients choose to tolerate pain in the face of pain. Chinese are also among the most likely to feel anxious about pain (54%), second only to the Russians (65%) and Japanese (62%), compared to the global average of 42%. Our group investigated 107 oral cancer patients and found that patients had high levels of catastrophizing beliefs and low levels of pain self-efficacy, while the results of a qualitative study [15] conducted at the same time also showed that some patients had a “fatalistic” view, showing a sense of helplessness and powerlessness that they could not fight against their fate. Chinese oral cancer patients generally have negative pain beliefs and tend to have negative feelings and perceptions about their pain experiences [15].

Studies [16–18] have confirmed that negative pain beliefs increase patients’ perception of pain and affect their attitudes and compliance with pain management. Al-Atiyyat [19] evaluated the attitudinal barriers to cancer pain management among adult Jordanian patients and to explore relationships between attitudinal barriers, pain, and demographic variables, concluded that the lack of positive pain attitudes and beliefs in cancer patients is currently the main barrier to poor pain control. Therefore, studies addressing interventions for pain beliefs have been conducted. Existing a study has mainly focused on the field of psychology and usually adopted a cognitive therapy approach to shape, modify, or replace beliefs, i.e., belief modification, which has shown that belief modification can regulate individuals’ irrational behaviors and the negative emotions they trigger and improve patient’ pain levels and quality of survival [20].

At present, the intervention methods of pain beliefs in patients with chronic pain include second-generation cognitive-behavioral therapy–Cognitive-Behavioral Therapy (CBT), which emphasizes changing cognition, and third-generation cognitive-behavioral therapy– Acceptance and Commitment Therapy(ACT), which is based on acceptance, of which third-generation cognitive-behavioral therapy is currently a hot topic of research in the related field [21–23]. Both CBT and ACT are cognitive behavioral therapy based on the fearing-avoidance model. The core part of cognitive remodeling is cognitive remodeling, and the basis of cognitive remodeling is belief revision [20], that is, in the process of psychotherapy, patients are guided to realize their own cognitive misunderstanding of pain through specific methods, and at the same time, patients are taught to choose the correct pain coping strategy. To help them cope actively and adapt to the pain. Studies [24, 25] have shown that acceptance strategies are one of the factors that determine patients’ psychological well-being and physiological functioning compared to altered cognition and pharmacotherapy. In the field of pain management, acceptance refers to allowing the pain experience to exist without making efforts to try to control the level of pain, but continuing with an open mind to live a normal life [26]. Acceptance is one of the revered concepts of Chinese Taoism, which fits with Eastern culture. The results of a previous study by our group showed that acceptance was the only pain belief-related indicator that could influence the degree of pain in oral cancer patients at different times [15]. Therefore, using pain acceptance as an entry point for belief revision-based pain management may help to reduce the pain level of oral cancer patients.

Leventhal’s common-sense model of self-regulation (CSM) is a common theoretical model in social psychology [27]. CSM is one of the theoretical frameworks for studying the pain beliefs of patients with chronic pain, which is formed from the perspective of cognitive science [28]. CSM suggests that individuals will act on their subjective guidance or common-sense perceptions of health threats (beliefs about illness/symptoms) and evaluate their effectiveness after implementation, and that the results will feed back into their cognitive and affective representations of illness. This in turn may influence the individual’s perception of illness and the choice of coping strategies. The model explains individuals’ perceptions of illness/symptoms and highlights the self-regulation of health and illness. The CSM is divided into cognition, response, and evaluation [27]. Cognition includes identity, consequences, cause, timeline, cure/control, and illness coherence six dimensions. The CSM suggests that individuals describe diseases according to the six dimensions. CSM is widely used in the health management of patients with cancer recurrence, rheumatoid arthritis, and gastroparesis, aiming to explore their health status such as medication compliance, illness perception, and delay in medical treatment [29–31]. But CSM is rarely used in oral cancer pain research.

In this article, we enrich how oral cancer pain belief modification program (CPBMP) was developed and examined by our research team, with reference for pain management of oral cancer patients.

## Methods

### Study design

A mixed-methods study was used to develop and revise the program. The CPBMP was developed and revised using the literature retrieval, semi-structured interview, and Delphi technique. The further improvement was explored with a one-group pre- and post-trial designed with a sample of 16 Chinese oral cancer survivors, and semi-structured interviews.

### Formulation of the CPBMP Draft

#### Literature retrieval

Electronic databases, including PubMed, Embase, CINAHL, Psych info, Cochrane Library, Web of Science, China National Knowledge Infrastructure (CNKI), Wanfang data, China Science and Technology Journal Database (VIP), Sino Med. Literature inclusion criteria: (1) age ≥ 18 years old cancer pain patients (P); (2) Any form(Online and/or offline interventions, One-on-one and/or group interventions) of cancer pain care based on belief modification (I); (3) Control group received routine cancer pain nursing (C); (4) Pain-related outcome indicators (O); (5) study types were randomized controlled trial (RCT) or clinical controlled trial (CCT) (S); (6) Languages ARE limited to Chinese or English. Literature exclusion criteria: literature about the belief modification combined with other measures; repeated published literature; full text of the literature was not available. The search time limit was from the establishment of the database to March 31, 2021. Using Medline as an example, the specific search strategy is shown in Table 1.

**Table 1:** The search strategy of RCT and CCT

**Electronic Databases**	**Search Strategy**
By Ovid sp Allied and Complementary Medicine Database (AMED), The search time limit: from the establishment of the database to March 31, 2021.	1 exp Neoplasms/ 2 exp "Squamous Cell Carcinoma of Head and Neck"/ 3 exp "Head and Neck Neoplasms"/ 4 exp Mouth Neoplasms/ 5 Neoplasia 6 Tumor.mp. 7 cancer.mp. 8 Malignancy.mp. 9 Malignant Neoplasm.mp. 10 Head and Neck cancer.mp. 11 oral cancer or mouth cancer.mp. 12 Oral Squamous Cell Carcinomas.mp. 13 Oral Cavity Squamous Cell Carcinomas.mp. 14 Laryngeal Squamous Cell Carcinomas.mp. 15 Hypopharyngeal Squamous Cell Carcinomas.mp. 16 Nasopharynx Squamous Cell Carcinomas.mp. **17 or /1—16.** 18 exp Health Education/ 19 exp Health Promotion/ 20 (health guid* or health instruct*).mp. **21 or /18—20.** 22 exp Pain Management/ 23 (pain intervention or pain nursing care or pain treatment or pain relie* or pain reduc* or pain control).mp. **24 or /22—23.** 25 (RCT or randomized controlled trial or controlled clinical trial or CCT).mp. 26 17and 21 and 24 and 25 27 limit 26 to English language

Two review authors Wang RN & Zheng XY according to the PICOs principle and inclusion exclusion criteria, independent retrieval and strict screening of literature are carried out. Studies ranked as irrelevant by both reviewers were excluded. In case of disagreement, it will be discussed and resolved. With a third (Gao J) resolving any disputes. Two review authors (Wang RN & Zheng XY) independently extracted data according to the data extraction form designed in advance, and then cross-checked the data, and checked the original text again to ensure the accuracy of data extraction.

Content analysis was used to analyze the literature. A total of 1 462 articles were retrieved according to the inclusion and exclusion criteria, including 709 English articles and 753 Chinese articles. Endnote X8 software was used to remove 348 articles, read 1 114 articles' titles and abstracts, excluded 1 021 articles that did not meet the title, read 93 full texts, and included 20 articles. After snowballing the included articles, 24 articles [32–55] were finally included. Two review authors(Wang RN & Zheng XY)evaluated according to JBI evidence pre-classification and evidence rank system for intervention research (2014 Edition) [56]. The pre-classification and evidence rank for included literatures were shown in Table 2.

**Table 2:** The pre-classification and evidence rank for included literatures (*n* = 24)

**Literature**	**Country**	**Time**	**Study Design**	**Quality level**	**Grade of recommendation**
Li [32]	China	2015	RCT	1c	A
Yang [33]	China	2020	RCT	1c	A
Zhu [34]	China	2016	RCT	1d	A
Guo [35]	China	2018	RCT	1c	A
Zhang [36]	China	2012	RCT	1d	B
Chen [37]	China	2014	RCT	1c	A
Fu [38]	China	2019	CCT	2d	A
Hu [39]	China	2007	RCT	1d	A
Li [40]	China	2013	CCT	1d	B
Sun [41]	China	2015	CCT	1d	B
Patsy [42]	Australia	2003	RCT	1c	A
Sandra [43]	USA	2009	RCT	1d	B
Mary [44]	USA	2012	RCT	1d	B
Yeur-Hur [45]	Taiwan, China	2014	RCT	1d	A
Yasemin [46]	Turkey	2009	RCT	1d	A
Barbara [47]	USA	1987	RCT	1d	B
Rianne [48]	Netherlands	2001	RCT	1c	B
Andreas [49]	Cyprus	2016	RCT	1c	A
Michèle [50]	Canada	2006	CCT	1d	B
Jennifer [51]	USA	2001	RCT	1d	B
Mimi [52]	Hong Kong, China	2012	RCT	1d	B
Tone [53]	Norway	2014	CCT	1d	A
Sandra [54]	USA	2008	RCT	1c	B
Sandra [55]	USA	2000	CCT	1d	B

#### Semi-structured Interview

Semi-structured, qualitative, in-depth interviews were conducted with eligible participants were in-patients who were initially diagnosed with oral cancer at a tertiary care hospital from March 2021 to May 2021. Purposive sampling was used to select patients who met the inclusion and exclusion criteria and were likely to provide rich information. The inclusion criteria were: (a) older than 18 years; (b) a Numerical Rating Scale (NRS) of at least 3 for chronic pain in the week prior to hospitalization; (c) undergoing a treatment plan of surgery; (d) having received a pathological diagnosis of oral cancer, coupled with an awareness of said diagnosis. Exclusion criteria included: (a) oral cancer recurrence or systemic metastases; (b) having a history of mental illness or currently taking psychiatric drugs; (c) having other diseases that cause pain; (d) being unable to communicate effectively due to disease or treatment; and (e) having previously participated in other research projects similarly.

The sample size was based on the repeated occurrence of interviewees' data and no new information appearing in the data analysis. The data reached saturation when the 15th patient was interviewed in this study and no new topics appeared, so the sample size was 15.

The interview schedule outlining which was based on the Common-Sense Model of Self-Regulation included (1) How do you feel about pain? (identity, consequences, cause, timeline, cure/control, and illness coherence); (2) What do you think when you pain? (consequences, emotional representations); (3) How do you deal with pain? (cure/control, pain coping responses); (4) How do you feel about the effects of pain management? (cure/control, pain coping responses). The interviews lasted 20 to 40 min. Colaizzi’s 7-step analysis method was used to extract three themes covering ten sub-themes: Pain Cognitive Representations of Oral Cancer, Pain Emotional Representations of Oral Cancer, and Pain Coping Responses. The ten sub-themes include pain identity, pain consequences, pain attributions, pain timelines, pain controllability/curability, and pain coherence, negative emotions, good pain resilience: building a new self, insufficient pain self-management, poor medical interaction.

### Revision of the CPBMP content

#### Delphi survey of the CPBMP

The CPBMP draft was revised by Delphi technique to evaluate the importance and feasibility for pain management in patients with oral cancer. Intentional sampling method was adopted in this study to select experts in oral and maxillofacial medicine, oral radiotherapy, pain and psychology. The inclusion criteria of experts in this study were as follows: (a) working in hospitals or medical colleges; (b) bachelor degree or above, intermediate or above title; (c) Doctors or nurses who have worked in the field of oral and maxillofacial surgery/pain/oral cancer radiation therapy for more than 10 years, or psychologists who have held the national psychological consultant Level 2 or above certificate and have been practicing for more than 8 years.

#### A feasibility experiment on 16 patients

The feasibility of CPBMP was explored with a simple single-group pre- and post-trial and semi-structured in-depth interviews. According to the study [50], the appropriate sample size for each group in the pilot study is 5–10 cases. Considering the withdrawal and loss of follow-up of patients during the study, the loss rate of follow-up in this study is prespecified to be 10%, so the sample size should be at least *n* = 10/ (1–10%) = 12 cases. Finally, 16 patients with oral cavity squamous cell carcinoma were recruited from the Department of Oral and Maxillofacial Surgery, The First Affiliated Hospital of Fujian Medical University in August 2021 to January 2023. The inclusion criteria for patients were as follows: (a) inpatients diagnosed with oral cavity squamous cell carcinoma; (b) 18 years of age or older; (c) NRS score of 3 points or greater; (d) patients undergoing surgery and radiotherapy; (e) primary school education or above, good communication and understanding skills; (f) clear consciousness, volunteer to participate in this study.

16 patients received CPBMP. The CPBMP is shown in Table 3. The pain management knowledge manual “Acceptance of Pain” for oral cancer patients was distributed for patients to avoid forgetting the details of intervention. Associate Professor of Nursing is responsible for project guidance and quality control of scientific research; the implement of psychological-related skills in the psychology teacher's guidance program; The doctor is responsible for answering the patient's questions about oral cancer and pain treatment; The head nurse is responsible for enhancing the patient's sense of trust, and asking and answering the patient's related physical, psychological and social questions from the perspective of the nursing staff; Graduate students and nurses in charge of oral and maxillofacial surgery are responsible for organizing, coordinating and implementing intervention programs, recording, feedback, collecting data, and collating and analyzing data. The study adopted a self-controlled design and was therefore unblinded to the intervention providers, participants, and outcome evaluators.

**Table 3:** The themes and content of the cancer pain belief modification program

**Themes of CPBMP**	**Content of CPBMP**	**Intervention Time/Duration**
1 Establish cancer pain files	1.1 Establish contact with study participants and assist patients/families to join the cancer pain management group WeChat group 1.2 Distribute and instruct patients in the use of pain management knowledge booklets 1.3 Fill out an oral cancer pain file	Day 1 of admission; 1–1.5 h
2 Understanding pain to avoid cognitive misunderstanding	2.1 Carry out health education on oral cancer pain 2.1.1 characteristics of oral cancer pain 2.1.2 effects of oral cancer pain 2.1.3 Clinical management of oral cancer pain 2.2 Focus on pain automatic thinking 2.2.1 pain automatic thinking definition 2.2.2 common pain automatic thinking (for example, pain is caused by a tumor, if the tumor is removed, there will be no more pain. Pain makes you irritable) 2.2.3 through communication to understand the patient's pain automatic thinking, health education for misunderstandings 2.3 focus on pain beliefs 2.3.1 pain beliefs define 2.3.2 common pain perceptions (such as: fatalism. Persistent pain can affect physical functioning, family roles 2.3.3 Correlation between pain beliefs and pain levels (for example, negative pain beliefs will increase pain levels) 2.3.4 understand patients' pain beliefs through communication and carry out health education for misunderstandings	Day 2 of admission; 1–1.5 h
3 Rational views of analgesics	3.1 Carry out health education on drug analgesia 3.1.1 commonly used analgesics for oral cancer 3.1.2 adverse reactions of common analgesics	Day 3 of admission; 1–1.5 h
4 Accept pain	4.1 Introduce the non-drug analgesia method of accepting pain to the patient and implement it on the spot to understand the patient's feeling after practice 4.1.1 Metaphor (Day 4) 4.1.2 Mindfulness breathing (Day 4) 4.1.3 Body scan (Day 5) 4.1.4 Mindfulness meditation (Day 6) 4.1.5 Music Therapy (Day 6) 4.1.6 Progressive muscle relaxation training (Day 7) 4.2 Encourage patients to keep practicing during the recovery period	Day 4–7 of admission; 1–1.5 h
5 Discharge guidance	5.1 Retrospective exercise 5.2 Pain self-report 5.3 Practice acceptance in your life (e.g., positive thinking about brushing your teeth, positive thinking about bathing, etc.)	24 h before discharge and 24 h after radiotherapy; 1–1.5 h
6 Summary feedback	6.1 Asking about practice and feelings, consulting and answering questions, consolidating communication	7 days, 1 month after surgery, 1 month after radiotherapy; 45-60 min

### Data collection

The draft was evaluated by two rounds Delphi survey. The experts’ suggestions and opinions were collected by Expert Correspondence Questionnaire prepared according to the CPBMP draft content. The questionnaire was composed of four parts. The first part was the instructions of the questionnaire, including the content and purpose of the questionnaire, the requirements for filling in the questionnaire, the way to collect the questionnaire, the time and the acknowledgements. The second part is the questionnaire of basic information of experts, which mainly analyzes the authority degree of experts, involving the basic personal information such as profession, professional title and age. The third part was the main body of the questionnaire, which was mainly aimed at the importance and operability of each item in the pain management plan and revised. Each item was set up with a column of "modification opinions", so as to understand the modification opinions and relevant basis of experts for each item and improve the reliability of expert consultation. The fourth part is the expert judgment basis questionnaire and the familiarity degree questionnaire to understand the experts' familiarity with the content of this study questionnaire and the relevant judgment basis. According to the experts' preferences and actual situation, we choose to communicate with experts by face-to-face letter consultation and email.

The authority of experts is expressed by the authority coefficient (Cr). The authority coefficient of experts is mainly determined by the familiarity of experts with the problem and the judgment basis of experts on the problem, which are expressed as Cs and Ca, respectively. (Cr) = (Ca + Cs)/2. It is generally believed that when the degree of expert authority (Cr) reaches 0.7 or above, the research results can be considered reliable and the research results are credible [57].

The basic data of patients was collected by the pain files of oral cancer patients, which consists of the general information questionnaire and the pain file of patients at different treatment stages, Study measures included Numeric Rating Scale (NRS), Chinese version of Illness Perception Questionnaire-Revised for Cancer Pain (IPQ-CaCP), and the University of Washington Quality of Life assessment scale (UW-QOL), which were conducted by a trained full-time master of nursing graduate student within 24 h of admission (T1), 24 h before discharge (T2), 24 h after the end of radiotherapy (T3), and 1 month after the end of radiotherapy (T4).

### Numeric Rating Scale (NRS)

NRS is the most widely used single-dimension assessment scale [58]. A straight line was divided into 10 equal parts, and each point was indicated by the number 0 to 10, with 0 as no pain and 10 as severe pain, according to the patient's self-evaluation.

### Chinese version of Illness Perception Questionnaire-Revised for Cancer Pain (IPQ-CaCP)

The questionnaire was translated by Guo Shuliu into a self-revised version of the Disease Cognition Questionnaire (IPQ-R) and the Disease Cognition Questionnaire for Slow Pain (IPQ-CP) [59, 60]. It consisted of seven dimensions, timeline acute/chronic beliefs, timeline beliefs, consequences beliefs, emotional representations, personal control beliefs, treatment control beliefs, and illness congruence, with a total of 38 items. Among them, timeline acute/chronic beliefs, timeline beliefs, consequences beliefs, and emotional representation were negative beliefs, and personal control beliefs, treatment control beliefs, and illness coherence were positive beliefs. Likert 5-point scoring method was used for each item, ranging from 1(strongly disagree) to 5(strongly agree), with a total score of 38 to 190. The higher the negative belief score, the more belief that cancer pain is chronic and cyclical, the more negative impact of cancer pain, and the more negative emotional manifestations. A higher positive belief score indicates a higher level of treatment control, a stronger personal belief in control, and a greater understanding of cancer pain. The Cronbach's ɑ coefficient of the Chinese version of the scale was 0.74.

### University of Washington Quality of Life Questionnaire, UW-QOL)

The scale consisted of two parts. The first part included 12 subjective evaluation items related to the disease, including vitality, pain, mood, shoulder function, appearance, anxiety, entertainment, saliva, taste, chewing, speech, and swallowing, which covered 12 problems that patients with head and neck cancer often faced in life. The second part contains three comprehensive questions; In addition, patients chose the items that they thought had the greatest impact on their life. According to the Likert scoring method, the scores of individual items range from 0(very poor) to 100(excellent), and the total score is the sum of each item /12. The score ranges from 0 to 100, and the higher the score, the better the recovery of the patient and the higher the quality of life [61]. We used the first part.

The semi-structured interview was used to understand the patients’ views on CPBMP after intervention. The interview outline includes (a) Do you think CPBMP can help you? Please be specific. (b) What do you think of the overall arrangement of the CPBMP? (c) What do you think are the shortcomings of the CPBMP? What improvements can be made?

### Data analysis

Data were analyzed using SPSS Statistics for Windows, version 24.0 (IBM Corp.). Descriptive statistics were used to describe demographic data. We used t-test to analyse the pain level, pain beliefs, and quality of life scores, before and after the intervention. The Shapiro–Wilk test was used to test the normality of the total score of patient outcomes and its dimension score before and after the intervention. If the measurement data follow a normal distribution and satisfy the homogeneity of variance, the t-test of two independent samples is used; If the normal distribution is not followed, the Mann–Whitney U-rank sum test is used. Qualitative content analysis was performed to analyse the qualitative data.

## Results

### CPBMP Draft

Through literature research, it was found that the content and methods of pain education were diverse, focusing on the knowledge education of drug analgesia and non-drug analgesia and pain management barriers. The methods included offline oral education, pain knowledge manual education, video education, photo album education and education based on a certain media (such as WeChat, telephone). Most studies used pain management manuals.

The qualitative study also found that oral cancer patients generally had negative pain beliefs, but there were still positive pain beliefs. It is suggested that medical staff should pay attention to the existence of patients' pain beliefs in clinical work, especially the influence of negative beliefs, and take pain acceptance as the entry point to carry out systematic and effective pain management, so that patients' pain control can achieve the desired effect.

However, no pain management scheme based on a complete theoretical framework has been constructed, and Yang’s studY [13] showEd that negative pain beliefs can affect the level of pain and the effect of pain management. At present, there are few intervention programs for pain beliefs. Based on the discussion of the research group, the CPBMP was constructed on the basis of literature research with belief revision as the main intervention measure, combined with drug analgesia and non-drug analgesia related health education content. The CPBMP draft is consisted of “Establish cancer pain files”, “Understand oral cancer and cancer pain”, “Treat analgesics rationally”, “Avoid cognitive misunderstanding”, “Accept pain”, “Start from value”, “Discharge guidance”, “Summary feedback”.

### Demographic characteristics

#### Experts’ Characteristic

A total of 15 specialists were included, including 7 cancer care specialists, 2 pain care specialists, 1 pain specialist, 1 psychotherapist, and 4 oral and maxillofacial surgery specialists. The result of experts’ demographic characteristics is shown in Table 4.

**Table 4:** Demographic characteristics of experts

**Number**	**Age**	**Education Degree**	**Professional Title**	**Major Direction**	**Years of Working**
1	40–49	Bachelor's degree	Associate chief nurse	Oncology Nursing	29
2	50–59	Bachelor's degree	Associate chief nurse	Oncology Nursing	36
3	40–49	Bachelor's degree	Associate chief nurse	Oncology Nursing	23
4	50–59	Bachelor's degree	Chief Nurse Practitioner	Oncology Nursing	33
5	30–39	Master's degree	Lecturer	Oncology Nursing	11
6	40–49	Master's degree	Nurse in charge	Oncology Nursing	12
7	30–39	Master's degree	Psychologist II	Psychology	8
8	40–49	Master's degree	Associate chief physician	Oral Radiation Therapy	21
9	40–49	Doctor's degree	Associate professor	Oncology Nursing	23
10	40–49	Doctor's degree	Associate professor	Pain Nursing	15
11	50–59	Doctor's degree	Associate professor	Painology	27
12	40–49	Doctor's degree	Associate professor	Oral and Maxillofacial Medicine	20
13	30–39	Doctor's degree	Professor	Oral and Maxillofacial Medicine	12
14	40–49	Doctor's degree	Professor	Oral and Maxillofacial Medicine	22
15	40-49岁	Doctor's degree	Professor	Pain Nursing	24

### Patients’ Characteristic

#### Baseline data, adherence and attrition

**Fig. 1:**
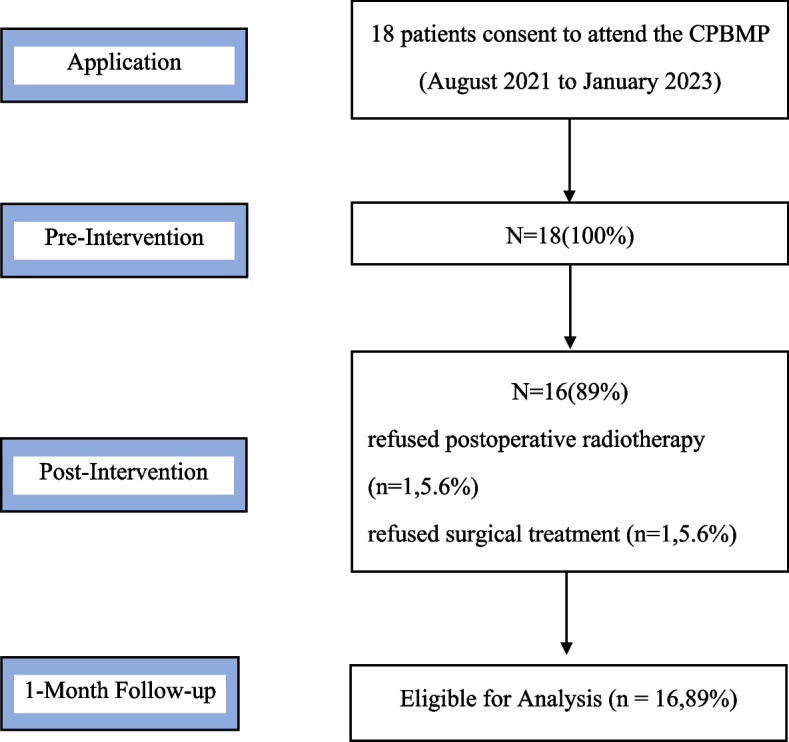
Participant Flow from Application to 1-month Follow-up

16 patients finished the intervention. The oral cancer patients ranged in age from 32 to 74, with an average age of (53.00 ± 13.09) years. The demographic of the sample is shown in Table 5. Specific details of participant flow, treatment attrition, lesson completion and questionnaire response are shown in Fig. 1. Post-treatment data were available for 89% (16/18) of participants and 89% (16/18) provided data at 1-month follow-up.

**Table 5:** Demographic characteristics of the patients

Variables	Sample size (*N* = 16, %)
Sex	
Male	13 (81.25)
Female	3 (18.75)
Marital status	
Married	15 (93.75)
Widowed	1 (6.25)
Education	
Primary school and below	2 (12.50)
Junior high school	4 (25.00)
High School	4 (25.00)
College	2 (12.50)
Bachelor degree or above	4 (25.00)
Clinical staging	
Phase I	2 (12.50)
Phase II	7 (43.75)
Phase III	2 (12.50)
Phase IV	5 (31.25)
Career	
Incumbency	8 (50.00)
Retirement	2 (12.50)
Unemployment	6 (37.50)
Place of residence	
Countryside	5 (31.25)
Township	2 (12.50)
County	3 (18.75)
City	6 (37.50)
Monthly income per household	
3000 ~ 4000	1 (6.25)
4000 ~ 5000	8 (50.00)
5000 ~ 6000	5 (31.25)
6000 ~	2 (12.50)
Health care status	
Out of pocket	2 (12.50)
Provincial medical insurance	1 (6.25)
Municipal health insurance	5 (31.25)
New rural Cooperative Medical System	8 (50.00)
Diagnosis	
Tongue cancer	10(62.50)
Gum cancer	1 (6.25)
Cheek cancer	3 (18.75)
Oropharyngeal cancer	2 (12.50)

#### The expert authority coefficient

The expert authority coefficient value was 0.75 in the first round of the Delphi survey and 0.78 in the second round.

### The first round of the experts’ suggestions for improving the CPBMP

Deleted “Start from value”.

Patients usually only have intuitive feelings or superficial cognition of diseases or symptoms, and it is difficult to raise their cognition to the level of value. (expert 2, expert 3).

Added “Focus on pain automatic thinking”.

Intervention with pain beliefs should probably be gradual and therefore added “Focus on pain automatic thinking”. (expert 6).

Merged Items with Overlapping Content.

The contents of “Think rationally about analgesics” “Understand oral cancer and cancer pain”, and “Avoid cognitive misunderstanding” were overlapped. (expert 12) Considering the slight differences in emphasis and operability of the items, they were merged into “Understanding pain to avoid cognitive misunderstanding” and “Rational view of analgesics”.

### The second round of the experts’ suggestions for improving the CPBMP

In the second round, the experts made no recommendations. We collated and analyzed the data, combined the entry screening threshold value method, and finally did not make any changes.

**Fig. 2:**
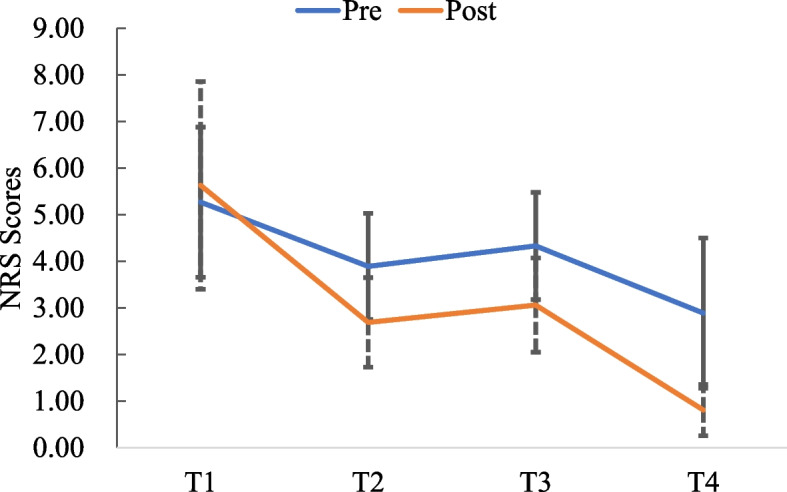
Pain intense scores at pre- and post-test

**Fig. 3:**
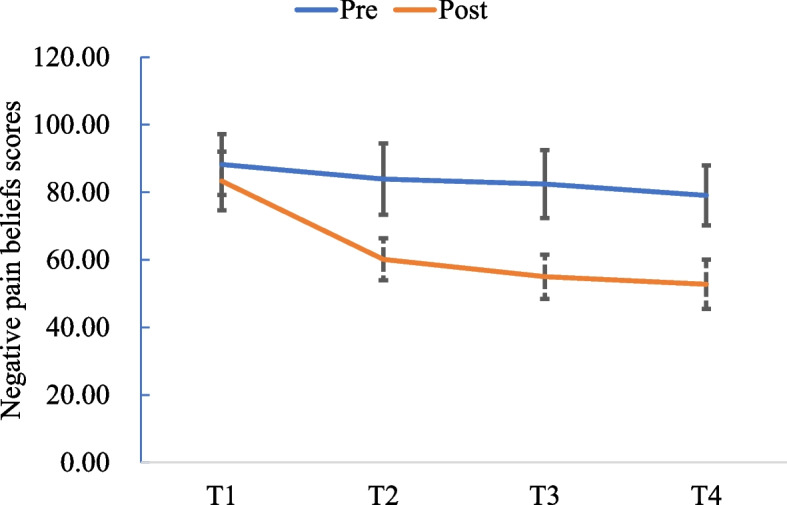
Negative pain beliefs scores at pre- and post-test

**Fig. 4:**
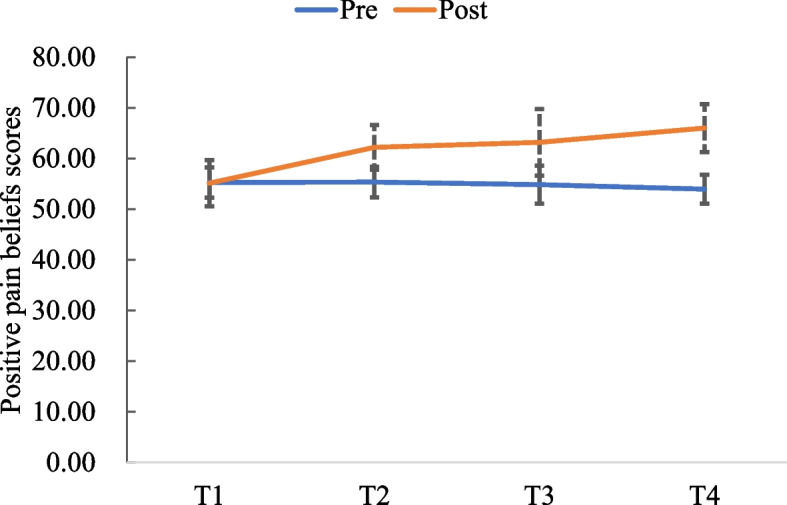
Positive pain beliefs scores at pre- and post-test

**Fig. 5:**
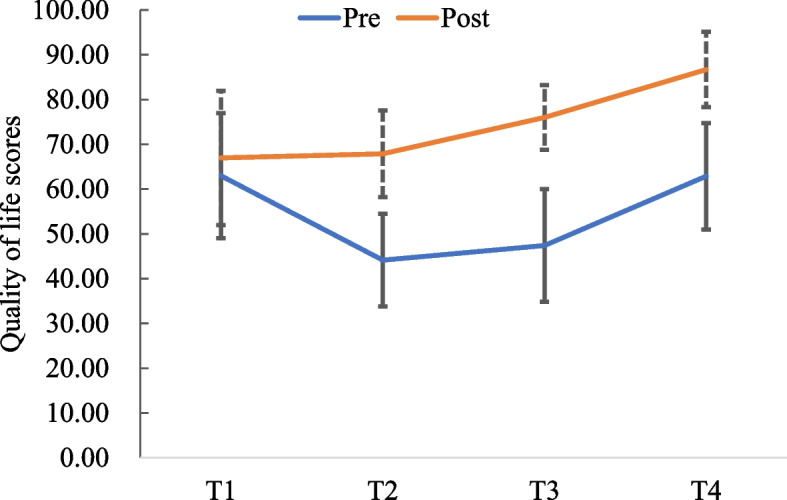
Quality of life scores at pre- and post-test

The pain intense, pain beliefs, and quality of life scores for the 16 patients (see Fig. 2, Fig. 3, Fig. 4, Fig. 5)

The average pain intense score at pre- and post-testing decreased from 5.63 ± 0.48 to 0.81 ± 0.54 (*t* = -3.746, *p* < 0.001). The average negative pain beliefs score decreased from 140.63 ± 9.02 to 52.75 ± 7.27 (*Z* = 12.406, *p* < 0.001). The average positive pain beliefs score increased from 55.13 ± 4.54 to 66.00 ± 4.70 (*Z* = -6.983, *p* < 0.001). The average quality of life score increased from 66.97 ± 15.01 to 86.69 ± 8.42 (*Z* = 7.283, *p* < 0.001).

### Experience of patients who have finished the intervention

#### Endorsed the CPBMP

Patients endorsed the CPBMP, and believed that CPBMP intervention could help them and regulate their own state. (Participant 3): “There is a lot of content in this booklet, which is what we want to know. It also teaches us how to accept the pain, which is good, and learning knowledge, and not so painful, the whole person becomes better.”

#### The overall arrangement structure is reasonable

Patients could accept the overall arrangement of the CPBMP, which was reasonable and meaningful. (Participant 5): “The overall arrangement is quite good, it includes pictures, audios, pamphlets, I think it’s quite good. I always felt boring after being sick, there’s nothing to do at home, so I like the intervention. It’s relaxing to practice, and doesn't take a long time.” (Participant 8): “It is simple, and don’t need me to do something complex. I just scan the two-dimensional code to listen to it. I felt quite relaxed. It takes my mind off things and helps me fall asleep faster.”

#### Retention Time Point: 24 h after radiotherapy

During and after the intervention, the feedback and suggestions of the patients on the CPBMP were obtained. They thought that the number of interventions could be appropriately reduced. (Participant 2): “There’s so many practices that sometimes we don’t have time to do this.” Patients suggested cancelling the questionnaire filling within 24 h of radiotherapy hospitalization to reduce bias. (Participant 7): “I did the questionnaire after surgery, and then went to the radiotherapy department to do it again after half a month’s rest. In fact, I still remember what I filled in last time. I think the interval is too short. I advise that we should cancel the questionnaire filling within 24 h of radiotherapy hospitalization.” After discission with our research team, we didn’t have to accept the advice, because the questionnaire filling was for measurement of the efficacy of the intervention but not the content of CPBMP.

## Discussion

### The necessity of the CPBMP

The oral cavity is an important functional part of the body, the distribution of blood vessels and nerves is rich, and the feeling is more sensitive. Therefore, oral cancer patients often suffer from severe chronic pain [3]. In the later stage, due to the influence of disease progression and related treatment, oral and maxillofacial pain is often persistent and increasing, which seriously affects the speech, swallowing and chewing functions of patients [62]. Therefore, it has become the most serious and concerned problem of patients with oral cancer [5, 63]. Persistent pain distress often leads to decreased treatment efficacy [64] and even loss of treatment compliance [65]. Studies [66] have confirmed that patients with positive pain beliefs can better deal with pain and its adverse effects. However, ignoring pain beliefs is an important factor affecting the effectiveness of pain management [67]. Therefore, this study based on the CSM model as the theoretical guidance, constructed CPBMP for patients with oral cancer based on belief revision. CPBMP involve six dimensions “Establish cancer pain files”, “Understand pain to avoid cognitive misunderstanding”, “Rational view of analgesics”, “Accept pain”, “Discharge guidance”, and “Summary feedback”.

Pain assessment is the basis of pain management. We help patients to establish a pain file in order to fully and dynamically understand the patient’s pain and ensure the development of pain management. We modify the patients’ pain beliefs through belief revision exercises, so as to improve the patients’ acceptance of pain and treat pain with a non-judgmental attitude, thus reducing the level of pain perception, effectively relieve pain and unpleasant emotions caused by pain, and make patients calmer. “Understand pain to avoid cognitive misunderstanding”, and “Accept pain” reflect the importance of paying attention to the beliefs and behaviors of patients with oral cancer pain, and enrich the belief research in the nursing field. CPBMP provides a variety of practice skills for pain acceptance, which is convenient for patients to practice according to their actual situation. In order to meet the continuity of patients’ health management needs, the plan cycle was extended from preoperative to one month after the standardized treatment of oral cancer to achieve the integrity of the management plan and improve the effectiveness and satisfaction of patients’ pain management.

### The acceptability of the CPBMP

Our findings support the acceptability of intervention. In the initial intervention phase, 18 entered the recruitment, and of those, 16 completed the intervention. Two participants withdrew; one due to not receiving postoperative radiotherapy, one owing to refused surgical treatment. The rejection rate of 2/18 (11.1%) may have resulted from the common phenomenon of most patients’ treatment decision-making dilemmas, rather than the attitude and beliefs of pain. During the intervention phase, patients agreed with CPBMP, and believed that CPBMP intervention could help them, reduce their pain, and regulate their own state. 

Patients could accept the overall arrangement of the CPBMP, which was reasonable and meaningful. 

In the CPBMP intervention, the Acceptance of Pain manual introduced the main content of each intervention module in simple language and related pictures, provided knowledge related to oral cancer and pain, which was convenient for the study subjects to understand their own disease condition, and helped the intervener to connect and transform different intervention modules. The omission of intervention content and the diversion of topics by the subjects were avoided. At the same time, it is also conducive to the research object to recall the intervention content, correct their bad cognition, regulate the negative emotions and effects caused by pain, and carry out pain self-management, thereby improving the acceptability of CPBMP intervention.

### Reliability of construction method of the CPBMP

The selection of experts and the number of experts is the key to Delphi method. The research subject determines the selection of experts, the number of experts depends on the research scale, and the accuracy of evaluation and prediction improves with the increase of the number of experts [68]. It is generally believed that 15 to 50 experts are the most appropriate, and the number of experts is too small to obtain meaningful results [68]. Too much leads to prolonged research time, increased energy and material costs. In this study, the number of experts in the two rounds of consultation was 15 and 14, respectively, which were all from tertiary hospitals and universities in East China, with a certain representability. The experts involved in oral and maxillofacial medicine and nursing, tumor radiotherapy and nursing, pain medicine and nursing, psychology and other related fields, and paid attention to the combination of theory and practice. The proportion of research and practice experts was basically equal, which was helpful for a more comprehensive evaluation of the content of the program.

### The effectivity of the CPBMP

The results of this study showed that the pain level, pain beliefs, and the quality of life of patients improved after CPBMP intervention, and the difference was statistically significant. Pain belief modification intervention can improve the negative pain beliefs of patients, promote the positive pain beliefs, and reduce the pain intensity. It may be related to the following reasons. Firstly, our study uses the pain acceptance technique to modify and repair individual beliefs and belief systems to improve the pain level of patients. Researchers ask and question the unreasonable pain beliefs of patients with oral cancer to make them realize the unreasonable of their own cognition and think repeatedly. Secondly, we draw lessons from the acceptance commitment therapy (ACT) concept, encourage patients to practice the relevant techniques of acceptance of pain, and hold a non-evaluation attitude towards pain to improve the pain level of patients. Acceptance refers to the willingness of individuals to stay in contact with their own physical feelings, thoughts, and emotions without evaluating, following, avoiding, or changing them [69]. Studies [70, 71] have shown that the acceptance strategy is an effective coping technique to increase pain threshold and reduce pain level. In this study, through pain acceptance practice and feedback, patients’ negative pain beliefs such as catastrophizing and poor survival with pain and their accompanying emotional distress and behavioral reactions are constantly corrected, so that patients can re-understand cancer pain, modify their negative pain beliefs, maintain and promote their positive beliefs, and improve their acceptance of pain, so as to avoid individual assumptions and expectations of pain. Reduce intrusive thoughts and high alert state, and then improve the pain level, improve the pain management effect and satisfaction of patients. We used the form of exchange, communication, sharing, feedback, audio, pictures, education knowledge manuals, etc., to supplement pain-related knowledge, emphasize the correction of negative beliefs, help patients express pain, understand patients’ inner needs, improve their pain intensity and pain beliefs, and raise their cognition to the level of belief. Finally, it promotes their behavior change, and improve their quality of life.

This study has several limitations to consider. First, our sample size for the feasibility study is small. We chose 16 oral cancer patients for our study. Second, the time for follow-up is short. However, the main aim of our study was to develop the CPBMP and initially assess its feasibility in the target population, rather than to confirm its efficacy. To confirm its perceived value and further efficacy for oral cancer patients, we will conduct a rigorous RCT with a much larger sample size.

## Conclusions

This study developed a Cancer Pain Belief Modification Program for patients with oral cancer in China within the framework of the Common-Sense Model of Self-Regulation. A feasibility study was explored to confirm the acceptability of CPBMP. To the best of the authors' knowledge, this is the first study focusing on pain beliefs intervention in patients with oral cancer. It can provide ideas for the pain management of cancer patients.

## Authors’contributions

Wang RN completed the study design with Gao J and Zheng XY. Su XX conducted data analysis and Huang XY. interpreted results. Wang RN, Liu HJ and Guo YL contributed to data collection. The manuscript was drafted by Wang RN and edited and approved by Gao J. The author(s) read and approved the final manuscript.

## Data Availability

The datasets used and/or analysed during the current study are available from the corresponding author on reasonable request.

## References

[1] Rodríguez-Molinero J, Migueláñez-Medrán BDC, Puente-Gutiérrez C (2021). Association between oral cancer and diet: an update. Nutrients.

[2] Jie H (2021). 2019 China Cancer Registry Annual Report [M].

[3] Khawaja SN, Jamshed A, Hussain RT (2021). Prevalence of pain in oral cancer: a retrospective study. Oral Dis.

[4] Maulina TIA, Hardianto A (2017). The incidence of oral squamous cellcarcinoma (OSCC) and its relationship with orofacial pain in oralcancer patients in West Java Province, Indonesia. J Oral MaxillofacSurg Med Pathol.

[5] Epstein JB, Miaskowski C (2019). Oral pain in the cancer patient. J Natl Cancer Inst Monogr.

[6] Raja SN, Carr DB, Cohen M (2020). The revised International Association for the Study of Pain definition of pain: concepts, challenges, and compromises. Pain.

[7] Oh PJ, Kim YH (2012). Meta-analysis of spiritual intervention studies on biological, psychological, and spiritual outcomes. J Korean Acad Nurs.

[8] Jensen MPA, Turner JA, Romano JM (1991). Coping with chronic pain: a critical review of the literature. Pain.

[9] Christine Z, Natalie B (2002). Cancer pain and psychosocial factors: a critical review of the literature. J Pain Symptom Manage.

[10] He T. The correlation between pain and pain beliefs in cancer patients. Chin J Nurs，2011;46(09):909–11.

[11] Ma LL, Wang WL, Zhao LP (2013). Research progress in pain belief of cancer patients. J Nurs Sci.

[12] Schwartz DP, DeGood DE, Shutty MS (1985). Direct assessment of beliefs and attitudes of chronic pain patients. Arch Phys Med Rehabil.

[13] Yang WY, Jiang LL (2016). Correlationship study of pain intensity and pain beliefs among oral cancer patients. Nurs J Chin PLA.

[14] Zhang XL. More than 90% of Chinese people endure "Chinese-style" pain[EB/OL], The Beijing News, 2016–10–25. https://www.bjnews.com.cn/health/2016/10/25/420954.html?from=groupmessage&isappinstalled=0.

[15] Su XX. Research onpain beliefs and its influence on pain of patients undergoing oral cancer surgery [D]. Fujian: Fujian Medical University; 2021.

[16] Becker MH, Maiman LA (1975). Sociobehavioral determinants of compliance with health and medical care recommendations. Med Care.

[17] Shutty M, DeGoode DE, Hoekstra DM. Beliefs about pain and treatment outcome in chronic pain patients. New York: Poster presented at the 95th annual meeting of the American Psychological Association; 1987 .

[18] Williams DA, Thorn BE (1989). An empirical assessment of pain beliefs. Pain.

[19] Al-Atiyyat NMH, Vallerand AH (2018). Patient-related attitudinal barriers to cancer pain management among adult Jordanian patients. Eur J Oncol Nurs.

[20] Sun DY. The theory evoution and practice development of belief revision in the perspective of psychotherapy since the 20th[D]. Shanghai: Shanghai Jiao Tong University; 2019.

[21] Zhang Q, Wang SJ, Zhu HY (2012). Acceptance and commitment therapy (ACT): Psychopathological model and processes of change. Chin Ment Health J.

[22] Fashler SR, Weinrib AZ, Azam MA (2018). The use of acceptance and commitment therapy in oncology settings: a narrative review. Psychol Rep.

[23] Carson JW, Carson KM, Porter LS (2007). Yoga for women with metastatic breast cancer: results from a pilot study. J Pain Symptom Manage.

[24] Kemani MK, Hesser H, Olsson GL (2016). Processes of change in acceptance and commitment therapy and applied relaxation for long-standing pain. Eur J Pain.

[25] Scott W, Dalya A, Yu L (2017). Treatment of chronic pain for adults 65 and over: analyses of outcomes and changes in psychological flexibility following interdisciplinary Acceptance and Commitment Therapy (ACT). Pain Med.

[26] Mccracken LM, Vowles KE, Eccleston C (2005). Acceptance-based treatment for persons with complex, long standing chronic pain: a preliminary analysis of treatment outcome in comparison to a waiting phase. Behav Res Ther.

[27] Howard L, Linda C (1987). Behavioral theories and the problem of compliance[J]. Patient Educ Couns.

[28] Wang RN, Huang XY, Su XX (2023). A scoping review of pain belief research in chronic pain patients. Chin J Nurs.

[29] Brandes K, Mullan B (2014). Can the common-sense model predict adherence in chronically ill patients? A meta-analysis[J]. Health Psychol Rev.

[30] Woodhouse S, Hebbard G, Knowles SR (2018). Exploring symptom severity, illness perceptions, coping styles, and well-being in gastroparesis patients using the common sense model. Dig Dis Sci.

[31] Van der Elst K, De Cock D, Vecoven E (2016). Are illness perception and coping style associated with the delay between symptom onset and the first general practitioner consultation in early rheumatoid arthritis management? An exploratory study within the CareRA trial. Scand J Rheumatol.

[32] Li XF, Liu W (2015). Effects of WeChat intervention on medication adherence and pain intensity in cancer patients. Chin J Nurs.

[33] Yang Y, Wang SQ (2020). Effect of health education based on knowledge, belief and action model on prognosis of cancer pain patients. Chin J School Doctor.

[34] Zhu DX (2016). Application of cluster nursing combined with health education in standardized treatment of cancer pain. Today nurs.

[35] Guo SM (2018). Effect of systemic health educati onon treatment compliance and quality of life among patients with malignant tumor chemothrapy. Chin J Health Educ.

[36] Zhang HC. Effects of cognitive behavioral intervention on cancer pain in patients with malignant tumors. Int J Nurs. 2012;31(5):887–8.

[37] Chen FY (2014). Application of knowledge-attitude-practice model nursing health education in cancer pain patients. Chin J Prim Med Pharm.

[38] Fu L (2019). Comprehensive intervention study on compliance of home analgesic medication in patients with moderate and severe cancer pain. Chin J Pract Nurs.

[39] Hu LJ (2007). The application ofself - management on cancer pain patient. J Nurs Admin strat.

[40] Li QP (2013). Application of health education nursing path management in standardized treatment of cancer pain. Med Innov Chin.

[41] Sun J, Chen M (2015). Effect of cognitive intervention on analgesic medication compliance in cancer pain patients. Int J Nur.

[42] Yates P, Edwards H, Nash R (2003). A randomized controlled trial of a nurse-administered educational intervention for improving cancer pain management in ambulatory settings. Patient Educ Couns.

[43] Ward SE, Wang KK, Serlin RC (2009). A randomized trial of a tailored barriers intervention for Cancer Information Service (CIS) callers in pain[J]. Pain.

[44] Thomas ML, Elliott JE, Rao SM (2013). A randomized, clinical trial of education or motivational-interviewing-based coaching compared to usual care to improve cancer pain management. Oncol Nurs Forum.

[45] Lai YH, Guo SL, Keefe FJ (2014). Effects of brief pain education on hospitalized cancer patients with moderate to severe pain. Support Care Cancer.

[46] Yildirim YK, Cicek F, Uyar M (2009). Effects of pain education program on pain intensity, pain treatment satisfaction, and barriers in Turkish cancer patients. Pain Manag Nurs.

[47] Rimer B, Levy MH, Keintz MK (1997). Enhancing cancer pain control regimens through patient education. Patient Educ Couns.

[48] de Wit R, van Dam F (2001). From hospital to home care: a randomized controlled trial of a Pain Education Programme for cancer patients with chronic pain. J Adv Nurs.

[49] Charalambous A, Giannakopoulou M, Bozas E (2016). Guided Imagery And Progressive Muscle Relaxation as a Cluster of Symptoms Management Intervention in Patients Receiving Chemotherapy: A Randomized Control Trial. PLoS One.

[50] Aubin M, Vezina L, Parent R (2006). Impact of an educational program on pain management in patients with cancer living at home. Oncol Nurs Forum.

[51] Oliver JW, Kravitz RL, Kaplan SH (2001). Individualized patient education and coaching to improve pain control among cancer outpatients. J Clin Oncol.

[52] Tse MM, Wong AC, Ng HN (2012). The effect of a pain management program on patients with cancer pain. Cancer Nurs.

[53] Rustøen T, Valeberg BT, Kolstad E (2014). A randomized clinical trial of the efficacy of a self-care intervention to improve cancer pain management. Cancer Nurs.

[54] Ward S, Donovan H, Gunnarsdottir S (2008). A randomized trial of a representational intervention to decrease cancer pain (RIDcancerPain). Health Psychol Off J Div Health Psychol Am Psychol Assoc.

[55] Ward S, Donovan HS, Owen B (2000). An individualized intervention to overcome patient-related barriers to pain management in women with gynecologic cancers[J]. Res Nurs Health.

[56] The Joanna Briggs institute levels of evidence and grades of recommendation working party supporting document for the Joanna Briggs institute levels of evidence and grades of recommendation. Australian: The Joanna Briggs Institute; 2014.

[57] Cheng C. Construction of ICU nursing quality sensitivity index system[D]. Anhui: Anhui Medical University: 2018.

[58] Zhang CH, Xu LH (2006). Pain assessment and care. Contin Med Educ.

[59] Guo S-L. Influence of beliefs about cancer pain and analgesics on pain experience outcomes in Taiwanese patients with lung or colorectal cancer[D]. Canada: University of Toronto; 2014.

[60] Moss-Morris R, Weinman J, Petrie K (2002). The Revised Illness Perception Questionnaire (IPQ-R). Psychol Health.

[61] Chen ZA. Retrospective study on the quality of life of patients after two skin flaps repairing oral and maxillofacial defects [D]. Nanchang: Nanchang University; 2020.

[62] Lam DK, Schmidt BL (2011). Orofacial pain onset predicts transition to head and neck cancer. Pain.

[63] Wang CH, Lee SY (2015). Undertreatment of cancer pain. Acta Anaesthesiol Taiwan.

[64] Zhang H, Meng J, Wang Q (2016). Effects of social support and treatment efficacy on coping style and treatment compliance of oral cancer patients. Chin Med Herald.

[65] Xu L (2018). Application of physical and mental health management concept in the treatment of malignant tumor patients. J Tradit Chin Med.

[66] Wang M. Study on the relationship between pain belief, self-efficacy and coping strategies in patients with lumbar disc herniation. Shaanxi: Yan'an University; 2020.

[67] Dawson R, Sellers DE, Spross JA (2005). Do patients' beliefs act as barriers to effective pain management behaviors and outcomes in patients with cancer-related or noncancer-related pain?. Oncol Nurs Forum.

[68] Li YY, Chen WJ (2018). Application status quo of Delphy method in nursing management. Chin Nurs Res.

[69] Hayes SC, Bissett RT, Korn Z (1999). The Impact of Acceptance versus Control Rationales on Pain Tolerance. Psychol Rec.

[70] Kohl A, Rief W, Glombiewski JA (2012). How effective are acceptance strategies? A meta-analytic review of experimental results. J Behav Ther Exp Psy.

[71] Masedo AI, Esteve MR (2007). Effects of suppression, acceptance and spontaneous coping on pain tolerance, pain intensity and distress. Behav Res Ther.

